# Mucinous ovarian tumour presenting as a ruptured incisional hernia

**DOI:** 10.1308/003588412X13373405385818

**Published:** 2012-10

**Authors:** D Toomey, D McNamara

**Affiliations:** Beaumont Hospital, Dublin,Ireland

**Keywords:** Ovarian cancer, Hernia, Mucin

## Abstract

We describe an ovarian borderline tumour that presented as an acute deterioration in an incisional hernia secondary to intraperitoneal mucin accumulation. The differential diagnosis associated with hernial sac contents and options for opportunistic diagnosis are discussed. This case raises awareness of potential serious diagnoses that may be overlooked during emergent hernia repair.

Several eponymous hernias describe the variety of organs and their pathologies that can manifest in abdominal wall hernial sacs.^1^ However, the hernial neck is also a window into the peritoneal cavity that can aid the alert clinician in opportunistic diagnosis of distant intra-abdominal pathology. This case describes the diagnosis of an ovarian tumour in a patient presenting with a ruptured incisional hernia.

## Case history

**Figure 1:**
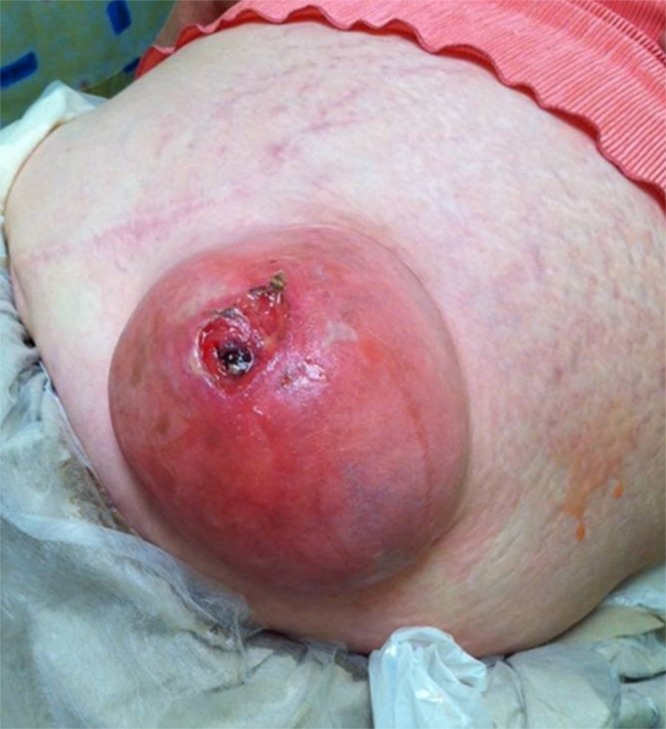
Incisional hernia (10cm) at the site of previous laparotomy with pressure necrosis of the skin. Mucin was seen to extrude through this defect.

A 61-year-old woman, with a 25-year history of a longstanding, lower midline, incisional hernia at the site of a previous appendicectomy, attended the emergency department complaining of a gelatinous discharge through a new skin defect overlying the hernia. The patient had no other significant signs or symptoms and, specifically, no clinical or radiological evidence of bowel obstruction or strangulation. On examination there was a 10cm spherical hernia in the left lower quadrant (Fig 1). Digital examination of the skin defect revealed palpable small bowel and omentum, and a thick viscous fluid was observed to extrude from the hernial sac. This finding prompted further investigation prior to embarking on surgical repair.

**Figure 2:**
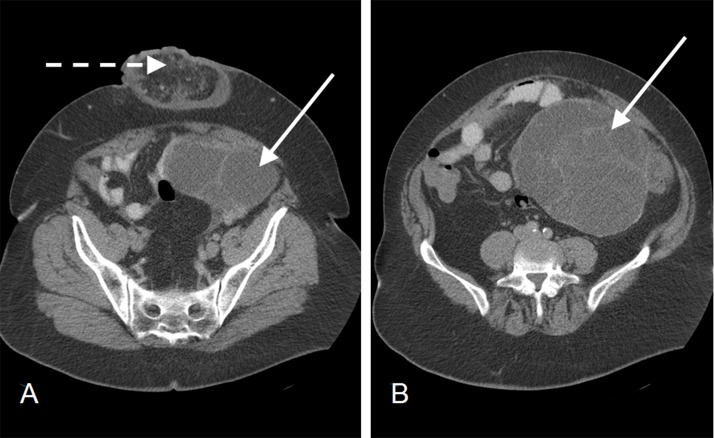
Computed tomography of the pelvis demonstrating a multiloculated mass (solid arrow) arising from the left adnexa and an incisional hernia containing omentum (dashed arrow)

Computed tomography (CT) revealed a 23cm multiloculated mass arising from the left adnexa (Fig 2). Serum CA19-9 was 786ku/l (normal: 0–35ku/l) and CA125 was 26ku/l (normal: 0–35ku/l). Cytological examination of the fluid was negative for malignant cells. The patient underwent a laparotomy and resection of the mass with an *en bloc* left oophorectomy. Histology was consistent with a borderline ovarian tumour of low malignant potential. Adjuvant chemotherapy was not recommended at a multidisciplinary team meeting. The patient made a good recovery and has been referred to plastic surgery for definitive management of the hernia.

## Discussion

A variety of organs and pathologies that may be encountered at hernia repair have been described including eponymous hernias such as Amyand’s (appendix) and Littre’s (Meckel’s diverticulum).^1^ Sacs containing epiploic appendagitis, a diverticular abscess, and various pelvic and gynaecological organs are also well reported.^2^

This case illustrates how the presence of unexpected fluid in a hernial sac led to the diagnosis of an ovarian neoplasm. Ascites is a not uncommon finding that, while often indicative of strangulation,^1^ can be associated with cirrhosis, malignancy or cardiac failure.^3,4^ Chylous ascites can be encountered in patients with thoracic duct injuries, cirrhosis, malignancy of the retroperitoneum or lymphatics, or mycobacterial disease. Blood may be due to haemoperitoneum from endometriosis, blunt trauma or a bleeding tumour.^3^ Faecal fluid or pus can be due to perforating or fistulating colonic disease. The presence of mucin should prompt a search for ovarian tumours, pseudomyxoma peritonei, an appendiceal carcinoma, a primary peritoneal tumour or mucinous tumours of the pancreas.^5^

Disruption of a hernia has been described previously in massive ascites.^4^ More commonly, increasing volumes of intra-abdominal fluid can raise the pressure in a ‘dormant’ hernia that may cause expansion, pain and erythema masquerading as strangulation, prompting emergent exploration.^4^ The alert surgeon, who may be focused on the prospect of a technically challenging, emergent reconstruction, should collect fluid samples for cytology when the opportunity arises, negating the need for further invasive sampling. If pre-operative CT has not raised the issue of an unexpected pathology, then extension of the hernia neck to permit a finger or hand may be useful. In appropriate circumstances, a laparoscope could be inserted through the hernia neck to visualise the peritoneum (hernioscopy) without further insult to the abdominal wall.^3^

## Conclusions

This case demonstrates an unusual presentation of an ovarian neoplasm that could easily be overlooked. We urge vigilance and awareness when tackling acute hernias that may conceal more sinister diagnoses.
